# Use of the λ Red-recombineering method for genetic engineering of *Pantoea ananatis*

**DOI:** 10.1186/1471-2199-10-34

**Published:** 2009-04-23

**Authors:** Joanna I Katashkina, Yoshihiko Hara, Lyubov I Golubeva, Irina G Andreeva, Tatiana M Kuvaeva, Sergey V Mashko

**Affiliations:** 1 Closed Joint-Stock Company "Ajinomoto-Genetika Research Institute", 1st Dorozhny Pr 1, Moscow 117545, Russia; 2 Fermentation and Biotechnology Laboratories, Ajinomoto Co, Inc, 1-1 Suzuki-cho, Kawasaki-ku, Kawasaki 210-8681, Japan

## Abstract

**Background:**

*Pantoea ananatis*, a member of the *Enterobacteriacea *family, is a new and promising subject for biotechnological research. Over recent years, impressive progress in its application to L-glutamate production has been achieved. Nevertheless, genetic and biotechnological studies of *Pantoea ananatis *have been impeded because of the absence of genetic tools for rapid construction of direct mutations in this bacterium. The λ Red-recombineering technique previously developed in *E. coli *and used for gene inactivation in several other bacteria is a high-performance tool for rapid construction of precise genome modifications.

**Results:**

In this study, the expression of λ Red genes in *P. ananatis *was found to be highly toxic. A screening was performed to select mutants of *P. ananatis *that were resistant to the toxic affects of λ Red. A mutant strain, SC17(0) was identified that grew well under conditions of simultaneous expression of λ *gam*, *bet*, and *exo *genes. Using this strain, procedures for fast introduction of multiple rearrangements to the *Pantoea ananatis *genome based on the λ Red-dependent integration of the PCR-generated DNA fragments with as short as 40 bp flanking homologies have been demonstrated.

**Conclusion:**

The λ Red-recombineering technology was successfully used for rapid generation of chromosomal modifications in the specially selected *P. ananatis *recipient strain. The procedure of electro-transformation with chromosomal DNA has been developed for transfer of the marked mutation between different *P. ananatis *strains. Combination of these techniques with λ Int/Xis-dependent excision of selective markers significantly accelerates basic research and construction of producing strains.

## Background

*Pantoea ananatis *belongs to the *Enterobacteriacea *family. The *P. ananatis *strain AJ13355 (SC17) was isolated from soil in Iwata-shi (Shizuoka, Japan) as a bacterium able to grow at acidic pH and showing resistance to high concentrations of glutamic acid [1]. These physiological features made this organism an interesting object for biotechnological studies, and for this reason its genome has been sequenced by Ajinomoto Co. (unpublished results). Nevertheless, up to the recent past, the absence of efficient genetic tools has hampered manipulations of this bacterium and retarded both basic research and applied investigations.

Over the last decade [2-7], the most powerful method for generating a wide variety of DNA rearrangements in *E. coli *has been termed "recombineering" (recombination-mediated genetic engineering) [8]. The term generally refers to *in vivo *genetic engineering with DNA fragments carrying short homologies with a bacterial chromosome, using the proteins of a homologous recombination system of the bacteriophage λ (λ Red system). Lambda *red *operon includes only three genes encoding Exo, Beta and Gam proteins. Gam inhibits the host nucleases, RecBCD and SbcCD, thereby protecting the dsDNA substrate for recombination [9,10]; Exo degrades linear dsDNA from each end in a 5'→3' direction, creating dsDNA with 3' single-stranded DNA tails [11-14]; and Beta stably binds a ssDNA greater than 35 nucleotides in length and mediates pairing the one with a complementary target [15-17].

The λ Red-mediated recombineering technology developed initially for modification of the genome of *Escherichia coli *K12 [2,4-7], was later broadened to other *E. coli *strains including enteropathogenic ones [18], and to *Salmonella *[19,20], *Shigella *[21,22], *Yersinia *[23,24], *Pseudomonas *[25], as well. Probably, one of the factors impeding application of this system in other hosts is the toxicity of expression of the λ Red genes for the cells.

In the present study, to overcome this toxicity for *Pantoea ananatis*, the special strain resistant to simultaneous expression of the λ Red genes was selected. Using this mutant, construction of all types of chromosomal rearrangements previously obtained in *E. coli *by recombineering was reproduced. The approach described may be used for adjusting the technology to other hosts.

## Results

### Construction of the new broad-host-range λ Red-expressing plasmid

To provide regulated expression of the λ Red genes in different bacteria, the plasmid pRSFRedTER [GenBank:FJ347161] based on the broad-host-range replicon of RSF1010 [26] has been constructed (see Additional file 1). This plasmid is useful for λ Red-mediated recombineering because: 1) the replicon is stably maintained in many Gram-negative [27] and some Gram-positive bacteria [28]; 2) the λ Red genes are placed under the control of the P_***lac***UV5 _promoter recognized by different bacterial RNA polymerases [29,30]; 3) the auto-regulated element P_***lac***UV5_-*lac*I provides IPTG-inducible expression of the λ Red genes with low basal level [31]; 4) the plasmid contains the levansucrase gene from *B. subtilis *allowing rapid and efficient recovery of this plasmid from the cells in a medium containing sucrose.

To test recombineering efficiency, pRSFRedTER mediated disruption of *galK *gene in *E. coli *MG1655 chromosome by integration of the PCR-generated DNA fragment carrying the Km^R ^gene from pUC4K flanked by *attL*_*λ *_and *attR*_*λ *_sites (*attL*_*λ*_*-*Km^R ^-*attR*_*λ*_) was performed. The constructed plasmid provided about 300 transformants per 10^8 ^survivors following electroporation. A similar frequency was obtained when pKD46 plasmid [4] was used as λ Red-expressing plasmid. In each case a chromosome structure of ten Km^R ^colonies was confirmed with PCR analysis.

Another plasmid carrying λ Red genes and named pRSFRedkan [GenBank:FJ347162], has been constructed via substitution of Cm^R ^and *B. subtilis sacB *genes by the Km^R ^gene from pUC4K [32].

### Concerted expression of λ Red genes is highly toxic for the *P. ananatis *wild-type cells

Clones of *P. ananatis *SC17 strain [1] obtained after electroporation by pRSFRedTER and plated on a solid LB-medium supplemented with Cm (50 μg/ml) were of very small size. Bacteria from these colonies grew very slowly in comparison with bacteria carrying pRSFsacB plasmid (see Additional file 1), which served as a vector for cloning of λ Red genes. Addition of IPTG (1 mM) for induction of expression of λ Red genes, led to complete cessation of the growth of pRSFRedTER containing cells. This effect was not detected for the cells carrying pRSFsacB and was based on the toxicity of the expression of λ Red genes.

To establish which component of the λ Red system caused the toxic effect, we constructed the pRSFGamBet [GenBank;FJ347163] plasmid lacking the *exo *gene encoding 5'→3' exonuclease.

It is well known that exonuclease activity is necessary for integration of dsDNAs only; integration of ssDNAs, such as chemically synthesized oligos, requires only recombinase (product of the *bet *gene, see [8]). To test the functional activity of the constructed pRSFGamBet, the plasmid was used to promote recombination between the artificial ss-oligos comprising two 36-nt homologies to the *galK *sequences and chromosome of the *E. coli *MG1655galK::(*attL*_*λ*_*-*Km^R^-*attR*_*λ*_) strain. As a result of recombination a native structure of *galK *gene was restored. Between (1.5–2.5) × 10^4 ^Gal^+ ^integrants per 10^8 ^survivors following electroporation were obtained in three independent experiments.

When introduced into *P. ananatis *SC17 strain, the pRSFGamBet plasmid did not inhibit cell growth even under the induced conditions (in the presence of 1 mM IPTG). Hence, the detected toxicity of pRSFRedTER was apparently caused by *exo *expression, or by simultaneous expression of all λ Red genes in *P. ananatis *cells.

### Selection of the recipient strain for λ Red-mediated recombineering in *P. ananatis*

A mutant *P. ananatis *strain, SC17(0), resistant to concerted expression of all λ Red genes, and thus manifesting the properties of a suitable recipient strain for λ Red-mediated integration of the linear DNAs into the chromosome, was obtained as follows. About 10 clones from 10^6 ^transformants obtained after electroporation of *P. ananatis *SC17 strain by pRSFRedTER, were of larger size after being plated on LB-agar with Cm. In LB-broth, bacteria from the "large" clones had a growth rate similar to the control strain with the pRSFsacB plasmid, and induction of the λ Red genes by IPTG caused only slight retardation in the growth of these cells.

Several of the selected "large" clones were cured from the plasmid on LB-agar containing sucrose, and re-transformed with pRSFRedTER. All clones grown after this re-transformation were of large size, similar to the parental clones. Three of the pRSFRedTER transformants that grew well were used as recipient strains for λ Red-mediated disruption of *hisD *gene. A PCR substrate, containing (*attL*_*λ*_*-*Km^R^-*attR*_*λ*_)-marker flanked by 40-bp homologous to the *hisD *gene, was electroporated into these strains. From 100 to 150 Km^R^His^- ^clones per 10^8 ^survivors following electroporation were obtained for each tested recipient strain. The insertion of the marker in the desired point of *hisD *gene was confirmed by PCR-analysis of 10 independent Km^R^His^- ^clones in each case. The observed integration frequency was similar to that obtained in the corresponding experiments with *E. coli *[4-6]. One of the plasmid-less strain used as a recipient in this experiment was named as SC17(0), and the obtained *hisD *strain constructed on its basis – as SC17(0)hisD::(*attL*_*λ*_*-*Km^R^-*attR*_*λ*_).

We tried to determine the nature of the mutation/mutations that provide resistance to concerted expression of λ Red genes to *P. ananatis*. There were no auxotrophic properties for SC17(0) strain growing on the M9 minimal media, supplemented with different carbon sources, in comparison with initial SC17 strain [1]. One of the possible explanations for the SC17(0) resistance is the reduced level of accumulation of λ Red proteins in this strain. To test the level of accumulation of λ Red proteins in SC17 and SC17(0), we performed SDS-PAGE of extracts of both strains carrying pRSFRedTER plasmid. Unfortunately, bands of λ Red proteins were not detected among the total cellular proteins even in conditions of IPTG induction for the both plasmid-carrier strains. The reduction in level of accumulation of λ Red proteins in SC17(0) strain, also, can be caused by decreased copy-number of RSF1010-replicon carrying plasmids in this strain. However, no reliable change in copy-number of pRSFsacB plasmid extracted from the cells of *P. ananatis *SC17 or SC17(0) strains could be experimentally found. On the other hand, the toxicity of the expression of λ Red genes has been detected for the plasmid-carrier cells of SC17 strain grown even without IPTG addition to the medium. In this case the transcription of the operon mediated by auto-regulated P_*lac*UV5_-*lacI *genetic element has to be tightly repressed. According to Skorokhodova et al. addition of 1 mM IPTG to the culture medium provides an increase of transcriptional level up to 10–20 fold [31]. But, the transcription level of λ Red genes under such conditions is not toxic for SC17(0). Hence, it is unlikely, that the synthesis of λ Red proteins in SC17 under the repressed conditions could be significantly higher than in SC17(0) after induction.

Perhaps, mutation/mutations that are present in the genome of the SC17(0) strain does not affect most of the genes encoding factors of global cellular regulation. At least, the patterns of total cellular proteins separated by 2D-PAGE in such a fashion that about 350 of individual polypeptides could be quantitatively analyzed [33], were not different for the SC17 and SC17(0) strains (data not shown).

Thus, the molecular mechanism of the resistance of the mutant SC17(0) strain to concerted expression of all λ Red genes is unknown as yet. Nevertheless, as will be shown below, various λ Red-driven modifications of bacterial chromosome could be provided on the basis of this selected strain.

### Use of the combined λ Red-Int/Xis system for introduction of multiple modifications

Earlier, a λ Int/Xis-driven system for removing the markers from *E. coli *chromosome was constructed, similar to one developed by Peredelchuk & Bennet [34,35]. It comprised of the plasmids carrying removable markers of Km^R ^or Cm^R ^flanked by *attL*_*λ *_and *attR*_*λ *_sites and the plasmid pMW-intxis-ts with thermo-sensitive pSC101-like replicon. This plasmid provided thermo-inducible expression of the *xis-int *genes under the control of λ P_R _promoter regulated by the temperature sensitive λ CIts857 repressor. Even being partially induced at 37°C, this system provided a high frequency (about 30%) of marker excision in *E. coli*. The high frequency of marker eviction at +37°C was very important for use of the system in *P. ananatis *because this bacterium has a lower temperature optimum than *E. coli *and cannot grow at 42°C (the standard temperature for temperature sensitive λ CIts857 repressor inactivation).

**Figure 1:**
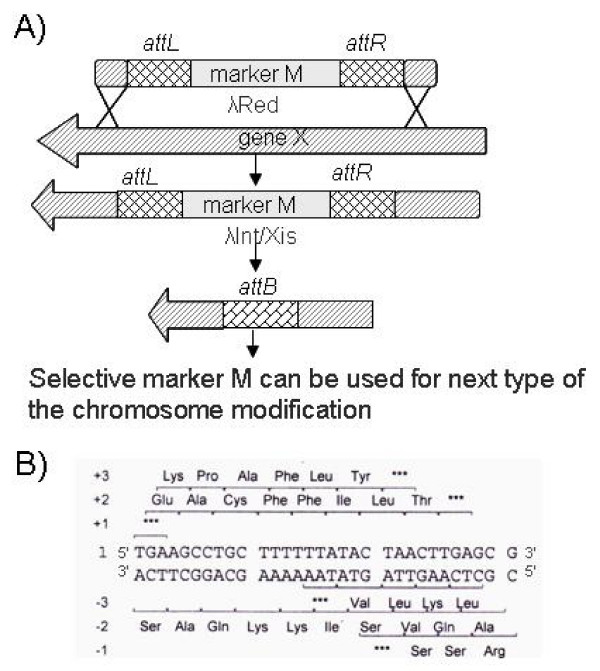
**Scheme of a construction of multiple chromosomal modifications, using the combined λ Red-Int/Xis system**. A) Selective marker M flanked by *attL*_*λ*_/*attR*_*λ *_is used for introduction of an appropriate mutation into the chromosome by λ Red-dependent recombination. Then the marker is eliminated from the chromosome by λ Int/Xis site-specific recombination. As a result, only the 31 bp long *attB*_*λ *_sequence linked to the mutation remains in the chromosome. Selective marker M can then be used in the next step of the introduction of multiple chromosomal modifications. B) The sequence of the *attB*_*λ *_site. One of the six ORFs provided by this sequence does not contain stop codons. Hence, it is possible to design an "in-frame" deletion of a gene. Asterisks mark stop codons.

The pMW-intxis-ts plasmid contains Ap^R ^gene as a selective marker that was not practicable for *P. ananatis *because of its high natural resistance to Ap. For this reason, we have substituted this marker with the Cm^R ^gene. The resulting plasmid (pMW-intxis-cat) was introduced to the SC17(0)hisD::(*attL*_*λ*_*-*Km^R^-*attR*_*λ*_) strain described above by electroporation. More than 30% of the transformants grown at 37°C on the plates containing LB-agar with Cm had lost the Km resistance. Loss of the Km^R ^cassette in the kanamycin-sensitive colonies was verified by PCR. Thus, the Km^R ^cassette can be used in the next step of the chromosomal modifications of this strain (Fig. 1A). Curing of the Km^S ^clones from the pMW-intxis-cat plasmid (with a frequency of 10%) was performed by re-streaking bacteria on LB-agar without antibiotic followed by cultivation at 37°C.

The *attB*_*λ *_site (31 bp in length), remaining in the chromosome after marker excision, contains six possible reading frames. One of these reading frames does not contain stop codons (Fig. 1B). Therefore, usage of the removable markers flanked with *attL*_*λ *_and *attR*_*λ *_sites allows design and construction of "in frame" deletions.

Using the λ Red-driven chromosomal modification followed by λ Int/Xis-mediated excision of the selective marker, it was possible to provide, step-by-step, the multiple chromosomal modifications in *P. ananatis *SC17(0) strain. This approach was repeatedly used for different modifications. Among them were 1) combinations of the simple or "in frame" deletions of several genes/operons; 2) integration of the marked heterologous genes into the chromosome of *P. ananatis *and 3) modification of the regulatory regions of the genes of interest [36]. Up to the present, several *P. ananatis *strains carrying more than 10 different modifications have been constructed using this strategy; the presence of multiple *attB*_*λ *_sites in their chromosomes did not hamper the repeated exploitation of this system.

### Transfer of marked mutations by electroporation of chromosomal DNA

General transduction is the most efficient and popular method for transfer of mutations between different *E. coli *strains. Although *P. ananatis *and *E. coli *are close relatives, the known *E. coli *transducing phages cannot infect *P. ananatis *cells. Therefore, development of another method for transfer of mutations between *P. ananatis *strains was necessary.

The electroporation of genomic DNA has been described for the transfer of genetic markers between different backgrounds of *E. coli *and *Pseudomonas *[37,38]. We tried to apply this technique to *P. ananatis*. The SC17(0)hisD::(*attL*_*λ*_*-*Km^R^-*attR*_*λ*_) strain was used as a donor of Km^R ^marker. Wild type strain SC17 was used as a recipient for electro-transformation of chromosomal DNA.

Previously, it was shown that special electroporation conditions are needed for the transformation of *E. coli *cells with large DNA molecules (see [39] for details). Different cultivation conditions of recipient strain and parameters of electroporation (electric field strength – E, time constant – τ) were tested. For *P. ananatis*, the highest yield of integrants (about 100 Km^R ^His^- ^integrants per 10^8 ^survivors following electroporation) was obtained under the following conditions. Recipient strain was grown up to absorbance of 0.8 – 1.0. Then electro-competent cells were prepared using 10 ml of culture as described in the "Plasmid electro-transformation" section (see "Methods"). Electroporation was performed at E = 12.5 kV/cm and τ = 10 msec (resistance of 400 Ω and capacity of 25 μF). Electro-transformation with chromosomal DNA is very fast method: all procedures, including DNA isolation and electroporation, can be performed in one day.

We found that marked chromosomal modification, obtained in *P. ananatis *SC17(0) strain via λ Red-recombineering method, could be easily transferred into the wild type SC17 strain by electroporation of chromosomal DNA. At the time of writing up to ten different mutations had been combined in the chromosome of the SC17 strain by repeated electro-transformation with chromosomal DNA followed by λ Int/Xis-driven excision of selective marker. The frequency of marked mutation transfer varied from several up to several hundreds of integrants per trial.

### Two-step λ Red-mediated introduction of unmarked mutations into *P. ananatis *chromosome

A two-step λ Red-mediated procedure for the introduction of unmarked mutations was elaborated for *P. ananatis *SC17(0). It comprises: 1) λ Red-driven insertion of dual selective/counter-selective marker into the desired point; 2) elimination of the marker via λ Red-mediated integration of the short dsDNA fragment containing the mutation of interest flanked with sites homologous to the appropriate target. Such an approach based on ET-recombination or λ Red-recombination was previously exploited for introduction of the unmarked mutations in *E. coli *[40,41]. One of the most popular counter-selective markers used for this purpose is the *sacB *gene from *B. subtilis*, whose introduction imparts sucrose sensitivity to the bacterium.

**Figure 2:**
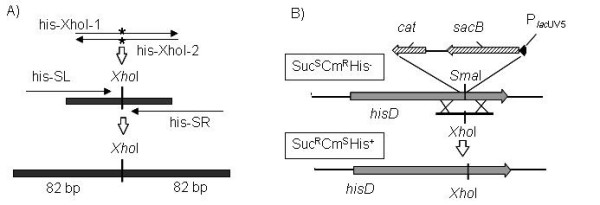
**Construction of the unmarked nucleotide exchange in the *P. ananatis hisD *gene**. A) Construction of a dsDNA fragment with appropriate mutation in the center. First, his-XhoI-1 and his-XhoI-2 oligos are annealed to each other. The nucleotide exchange of interest included in the sequence of his-XhoI-1/his-Xho2 olig is indicated by asterisks. The resulting dsDNA fragment is then used as DNA-template for PCR-amplification with his-SL and his-SR oligos. As a result a linear dsDNA fragment, harboring a *Xho*I restriction site and 82 bp long arms homologous to the target region of *hisD *gene, was obtained. B) The cassette containing dual selective/contra-selective marker is integrated into the target point of *hisD *gene. The constructed *in vitro *linear dsDNA fragment or ssDNA harboring appropriate mutation in the center is then integrated into the chromosome of this strain by the λ Red recombination system. As a result, the dual selective/contra-selective marker is eliminated from the chromosome with simultaneous introduction of the desired mutation into the *hisD *gene. This mutation leads to substitution of the native *Sma*I restriction site by the *Xho*I restriction site and restoration of the amino-acid sequence of HisD protein. Integrants are selected as colonies resistant to sucrose. Such colonies are subsequently tested for Cm sensitivity, ability for growth without histidine and presence of *Xho*I restriction site in the target chromosome point.

To create a template for the PCR amplification of the dual selective/counter-selective marker, *cat*/*sacB*, the pRSFPlacsacB plasmid was constructed. The cassette P_*lac*UV5_-*sacB*-*cat *was amplified with primers containing 36-nt homologies to the target on their 5'-ends, and integrated into *Sma*I recognition site located in *hisD *gene using SC17(0) harboring pRSFRedkan as a recipient and the *cat *gene in the cassette, as the selective marker. A short 170-bp long dsDNA fragment harboring an appropriate mutation and 82-bp long flanks homologous to the target region has been constructed (Fig. 2A). The desired modification of bacterial chromosome (substitution of the artificial *Xho*I for the native *Sma*I site) was finally achieved via the λ Red-mediated integration of the obtained DNA fragment accompanied by elimination of P_*lac*UV5_-*sacB*-*cat*, using *sacB *gene for counter-selection (Fig. 2B). The integrants of interest were found with a rather high frequency (25%) among the clones grown on LB-agar containing 30% sucrose.

This procedure could be applied for introduction of unmarked mutations to the genome of SC17(0) strain. Transfer of the obtained unmarked mutations to other strains by electro-transformation with chromosomal DNA is difficult because of impossibility of direct selection of integrants. Hence, to provide construction of unmarked mutations in the genome of the *P. ananatis *strains, sensitive to expression of all λ Red genes, we performed the method of λ Beta-driven integration of single-stranded oligos into the chromosome of SC17 strain.

### λ Beta-driven integration of single-stranded oligos

As mentioned above, ssDNAs, e.g. oligos containing short flanks homologous to the target site, can be integrated into the *E. coli *chromosome using only the product of the λ *bet *gene [8]. As the expression of *gam *and *bet *genes from pRSFGamBet plasmid was not toxic towards *P. ananatis *SC17 even under induced conditions, we tried to perform λ Beta-driven integration of oligos into the chromosome of this strain.

The *hisD*::P_*lac*UV5_-*sacB-cat *chromosomal modification was transferred into the SC17 strain by electro-transformation with genomic DNA isolated from SC17(0)hisD::P_*lac*UV5_*-sacB-cat *strain. The resulting strain, selected on LB medium supplemented with Cm, was named SC17hisD::P_*lac*UV5_*-sacB-cat*. The pRSFGamBet-kan [GenBank:FJ347164] plasmid was constructed by substitution of *sacB *and *cat *genes of pRSFGamBet for the Km^R ^gene from pUC4K. This plasmid was introduced to the obtained SC17hisD::P_*lac*UV5_*-sacB-cat *strain. It is known from the literature [8,42] that the efficiency of λ Red-dependent integration of the oligos depends on a direction of replication through the recombination site. The direction of replication at the *hisD *locus in the *P. ananatis *chromosome is not known. Hence, the plasmid-carrier cells were independently electroporated with hisD-XhoI-1 and hisD-XhoI-2 ss-oligos complementary to each other. Cells were plated on LB medium containing 30% sucrose. No colonies were observed after 24-hour cultivation when hisD-XhoI-2 olig was used for electroporation. About 200 clones were obtained after electro-transformation with hisD-XhoI-1 olig. To test the phenotype of the obtained transformants, 100 clones were replicated on the following solid mediums: LB medium supplemented with 30% sucrose, LB medium supplemented with Cm and M9 minimal medium. Seven of the tested clones had Suc^R^Cm^S^His^+ ^phenotype. These colonies were further verified by PCR and restriction of the amplified product. The presence of the expected mutation (substitution of the *Sma*I by *Xho*I recognition site) was confirmed in all of the seven Suc^R^Cm^S^His^+ ^clones.

Although the frequency of selection of the desired mutation was not high, in principle, λ Beta-dependent integration of ssDNAs allowed the construction of the unmarked mutations in the *P. ananatis*.

## Discussion

It is currently well established that high-frequency recombination between short homologies can be catalyzed in *E. coli *cells by the λ Red functions [4-7,43-45]. Unfortunately, up to the present, the range of bacteria for which this system has been utilized is limited. The main goal of the present study was to widen use of the λ Red-recombineering technology to *P. ananatis*, a bacterium of interest in the field of metabolic engineering. A broad-host-range λ Red-expressing plasmid useful for *P. ananatis *was constructed. The observed general toxicity of simultaneous expression of the λ *gam*, *bet*, and *exo *genes for *P. ananatis *SC17 cells was overcome by selection of the special recipient, SC17(0).

It is known that expression of λ Red functions in *E. coli *cells can lead to toxic effects [46,47]. Certainly, expression of λ Red genes has been investigated for *E. coli *in detail. In addition to its direct influence on traditional recombination pathways due to inhibition of RecBCD and SbcCD nucleases by λ Gam, the activity of λ Red proteins can interfere with the processes of replication and repair (see, [48,49] for reviews). For example, prolonged expression of *gam *gene could lead to formation of linear multimers of high, medium and low copy-number plasmids, and, even, of minichromosomes [50-53]. Expression of *exo *gene in addition to *gam *enhances this effect [50,52]. The plasmid linear multimers, also, may interfere with λ Red-recombination [18]. Murphy and co-workers showed that extended expression of λ Red-recombination functions could significantly induce a spontaneous mutagenesis, probably caused by interfering with mismatch repair UvrD-dependent pathway of *E. coli *[18].

Toxicity of expression of λ Red genes for the bacteria closely related to *E. coli*, but differing in the enzymes of replication, recombination and reparation, could not be predicted in advanced, as in the case of *P. ananatis *SC17. It is difficult, as well, to give an univocal explanation of how λ Red-mediated toxicity could be overcome. It seems that the decrease of this toxicity could be based on (*i*) lower intracellular level of λ Red proteins in the mutant cell, caused by reduced level in the synthesis of λ Red proteins or by increased efficiency of the specific proteolysis of λ Red proteins, (*ii*) decreased affinity of specific targets for interaction with λ Red proteins or increased level of biosynthesis of these targets. As for the *P. ananatis *mutant strain obtained, both variants of the explanation may be possible. As mentioned in the Results section, it is not very probable that SC17(0) strain possesses reduced level of the synthesis of λ Red proteins. But this possibility can not be completely rejected.

Nevertheless, even without information concerning the nature of the SC17(0) mutation, it is possible to use the corresponding strain for the desired λ Red-mediated rearrangements of *P. ananatis *chtomosome. All types of chromosome modifications constructed in *E. coli *by the λ Red-mediated recombineering, have been successfully reproduced in SC17(0) with pRSFRedTER as λ Red-expressing plasmid. Typically, the yield of recombinants varied from several tens to several hundreds per trial.

Using SC17(0) as the initial recipient for λ Red-promoting modifications it is subsequently possible to transfer the marked mutation to other *P. ananatis *strains of interest using the method of electro-transformation with chromosomal DNA. This method is a unique way to combine the set of marked mutations constructed in different *P. ananatis *strains into a single strain. In addition, potentially, co-transfer of rather closed mutations could be prevented by digestion of the chromosomal DNA by the appropriate restriction endonucleases, whose recognition sites are located between the mutations.

As the number of combined mutations of interest exceeds the number of available antibiotic resistance markers, curing of the intermediate strains from the used selective markers is necessary. Moreover, the presence of antibiotic resistance genes in the genomes of the industrial strains is rigorously restricted by the legislations of different countries. A wide variety of systems for marker curing based on the site-specific recombination systems (Cre/*lox*, Flp/*FRT*) are well-known [54-57]. These systems provide "symmetrical" recombination reaction between two identical sites flanking the removing marker, e.g. FRTxFRT = 2FRT. In this case, after removing the marker, the active site remains in the chromosome. Repeated action of such systems can lead to inversion or deletion of extended chromosomal fragments caused by site-specific recombination between the sites remaining at different points in the chromosome. Therefore, use of systems providing "asymmetrical" recombination reaction would be preferable. One such system is the site-specific recombination system of λ phage. It includes the Int and Xis proteins encoded by *int *and *xis *genes that, together with the host factors (IHF, RecA and Fis), provide the following reaction: *attL*_*λ*_x *attR*_*λ *_= *attP*_*λ*_+attB_*λ*_. Peredelchuk & Bennet [34] were the first who used this system for removal of the selective markers flanked by *attL*_*λ *_and *attR*_*λ *_sites. The *attB*_*λ *_site remaining in the chromosome after marker excision cannot recombine with *attL*_*λ*_or *attR*_*λ *_site in the next steps of strain construction. Thus, repeated action of the system would not influence the strain stability. Certainly, residual *attB*_*λ *_sites could recombine with each other via host general recombination system or provoke replication errors, especially if many left over scars (*attB*_*λ*_) are presented in chromosome and their positions were rather close to each other. Such events would lead to deletions of chromosomal fragments.

We adjusted the λ Int/Xis-dependent system for use in high-efficient marker excision in *P. ananatis*. Note that it is also possible, in particular, to design marker-less strains, carrying "in-frame" deletions.

Finally, the two-step procedure for introducing the unmarked mutations into the *P. ananatis *genome was demonstrated using *B. subtilis sacB *gene as a counter-selective marker. Desirable mutants were achieved at the second stage via λ Red-mediated integration of the short dsDNA in SC17(0) or ssDNA in any other *P. ananatis *strain carrying the dual selective/counter-selective marker in the target point of the genome.

Up to the present, the developed λ Red-mediated method has been used for deletion of more than 50 *P. ananatis *genes whose products were involved in central metabolism, respiration, transcription regulation, *etc*. Several marker-less *P. ananatis *strains carrying multiple (> 10) different chromosomal modifications including "in-frame" deletions, point mutations, rearrangements of regulatory regions, in particular, were constructed for basic research and applied purposes using combined application of the λ Red-Int/Xis systems and electro-transformation with chromosomal DNA.

## Conclusion

The λ Red-mediated recombineering has been adjusted for rapid and efficient construction of genome rearrangements in *P. ananatis*. In combination with the established procedures of λ Int/Xis-dependent marker elimination and electro-transformation with chromosomal DNA, this method provides a simple route to obtaining marker-less strains carrying multiple mutations of different types (deletions, substitutions of regulatory regions, integration of heterologous genes, and point mutations). The described approach of selection of the recipient strain resistant to expression of λ Red genes could be useful in exploiting λ Red-recombineering in other bacteria.

## Methods

### Strains and plasmids

Strains and plasmids used or generated in this study are listed in Table 1. A detailed description of plasmids obtained in this work is in Additional file 1.

**Table 1:** Strains and plasmids used or generated in this study.

Name	Main characteristics/accession number	Source or reference
Strains		
*Pantoea ananatis *SC17 (AJ13355)	mutant with decreased secretion of mucus	[1]
*Pantoea ananatis *SC17(0)	Derivative of SC17 resistant to expression of λ Red genes	This work
*E. coli *K12 MG1655	Wild type	VKPM

Plasmids		
RSF1010	GenBank accession number NC_001740	[26]
pUC4K	GenBank accession number X06404	[32]
pMW-*attL*_*λ*_*-*Km^R^-*attR*_*λ*_	Donor *attL*_*λ*_*-*Km^R^-*attR*_*λ *_cassette; Ap^R^; Km^R^	[35]
pKD46	pINT-ts; λ *gam*, *bet*, and *exo *genes are under P_***araB ***_promoter; Ap^R^	[4]
pRSFRedTER	λ *gam*, *bet*, and *exo *genes are under control of P-element; *sacB *gene; Cm^R^	This work
pRSFRedkan	λ *gam*, *bet*, and *exo *genes are under control of P-element; Km^R^	This work
pRSFGamBet	λ *gam *and *bet *genes are under control of P- element; *sacB *gene; Cm^R^	This work
pRSFGamBetkan	λ *gam *and *bet *genes are under control of P- element; Km^R^	This work
pRSFPlacsacB	P_lacUV5_-*sacB-cat *cassette; Cm^R^	This work
pMW-intxis-cat	pSC101-ts; λ *xis-int *genes transcribed from λ P_R _promoter under CIts857 control; Cm^R^	This work

A P-element marks the auto-regulated element P_***lac***UV5_-*lac*I [see [31] for details].

### Media and Growth conditions

*E. coli *and *P. ananatis *strains were cultivated with aeration in LB medium at 37°C and 34°C, respectively. The following antibiotic concentrations were used to select transformants and to maintain the plasmids: Km – 40 mg/l, Cm – 50 mg/l. The M9 salt medium supplemented with galactose (1 g/l) or glucose (1 g/l) was used to select Gal^+ ^or His^+ ^cells.

### Recombinant DNA techniques

DNA manipulations were performed according to standard methods [58]. Restrictases were provided by "Fermentas" (Lithuania). T4-DNA ligase was from Promega (USA). All reactions were performed according to the manufacturer's instructions. PCR was carried out with Taq-polymerase ("Fermentas"). Primers were purchased from "Syntol" (Russia). All primers used in this work are listed in Additional file 2.

### Construction of integrative cassettes

To provide cassettes for λ Red-dependent integration, the appropriate selective marker was amplified by PCR with oligos containing on their 5'-ends 36-nt sequences homologous to the target region. To disrupt *E. coli galK *and *P. ananatis hisD *genes, a removable Km^R ^marker flanked by *attL*_*λ *_and *attR*_*λ *_was amplified with galK-5/galK-3 and hisD-5/hisD-3 primers, respectively. The pMW-*attL*_*λ*_*-*Km^R^-*attR*_*λ *_plasmid was used as DNA template. To obtain insertion into the *P. ananatis hisD *gene, the P_lac_-*sacB*-*cat *cassette was amplified in PCR with his-Plac-5/his-cat-3 primers using pRSFPlacsacB plasmid as template.

### Construction of the linear dsDNA fragment to exchange native *Sma*I recognition site in the *P. ananatis hisD *gene by *Xho*I site

First, his-XhoI-1 and his-XhoI-2 oligos complementary to each other were annealed. Both oligos contained sequences corresponding to the *Xho*I restriction site in the center and arms homologous to the sequence surrounding native *Sma*I site of the *P. ananatis hisD*. As a result, the short dsDNA fragment containing the *Xho*I recognition site in its center and 33 bp long arms homologous to the target region, was obtained. The obtained fragment was amplified and extended by PCR with the primers his-SL and his-SR. The resultant DNA fragment generated by PCR contained *Xho*I recognition site in its center flanked with 82 bp arms homologous to the appropriate site in *P. ananatis hisD *gene.

### Plasmid electro-transformation

An overnight culture of *P. ananatis *strain grown at 34°C with aeration was diluted with fresh LB broth 100 times and the cultivation was continued up to the OD_**600 **_= 0.5–0.8. Cells from ten millilitres were washed three times with an equal volume of deionized ice water followed by washing with 1 ml of 10% cold glycerol and resuspended in 35 μl of 10% cold glycerol. Just before electroporation, 10–100 ng of the plasmid DNA dissolved in 2 μl of deionized water was added to the cell suspension. The procedure of plasmid electro-transformation was performed using the GenePulser and Pulse Controller ("BioRad", USA). The applied pulse parameters were: electric field strength of 20 kV/cm, time constant of 5 msec. After electroporation, 1 ml of LB medium enriched with glucose (5 g/l) was immediately added to the cell suspension. Then the cells were cultivated under aeration at 34°C for 2 h and plated on LB-agar containing the appropriate antibiotic. This was followed by an overnight incubation at 34°C. A competence of *P. ananatis *cells, determined for RSF1010 plasmid, was 10^6 ^CFU per μg of DNA. Typically, 10^5^–10^6 ^antibiotic resistant colonies were obtained per 10^8 ^survivors following electroporation.

### Gene rearrangement

Overnight cultures of *P. ananatis *or *E. coli *strains harbouring the plasmid, expressing appropriate λ Red genes, grown in LB broth with Cm (for pRSFRedTER, pRSFGamBet plasmids) or Km (for pRSFRedkan, pRSFGamBetkan plasmids) were diluted 100 times with the same fresh medium supplemented with 1 mM IPTG for induction of the λ Red genes. At culture density of 0.5–0.6, electro-competent cells were prepared as described above. From 200 to 500 ng of a PCR-generated linear dsDNA or 100 ng of a ss-oligos were used for transformation. Electroporation was carried out at electric field strength of 25 kV/cm and time constant of 5 msec for both types of DNA substrates. The chromosome structure of the obtained transformants was verified in PCR with galK-t1/galK-t2 primers for *E. coli galK *gene disruption, hisD-t1/hisD-t2 for *P. ananatis hisD *gene disruption and for insertion of the double selective/contra-selective marker into the *P. ananatis hisD*.

Gene disruption provided with pKD46 plasmid was performed as described in (4).

### *P. ananatis *electro-transformation with chromosomal DNA

Cells were grown in LB medium up to OD_**600 **_= 0.8–1.0. Electro-competent cells were prepared as described above. From 1 to 2 mg of a chromosomal DNA, isolated using a Genomic DNA Isolation Kit (Sigma), was used for transformation. Electroporation was carried out with an electric field strength of 12.5 kV/cm and time constant of 10 msec.

### SDS-PAGE

SDS-PAGE of cell extracts was performed according to Laemmli [59] with polyacrylamide gel with linear gradient of concentrations from 10% to 15%.

## Abbreviations

Ap: ampicillin; Ap^R^: ampicillin resistance; Cm: chloramphenicol; Cm^R^: chloramphenicol resistance; *cat*: chloramphenicol resistance gene; Km: kanamycin; Km^R^: kanamycin resistance; *kan*: kanamycin resistance gene; *sacB*: gene encoding the levansucrase; *attL*_*λ*_: *attL *site of phage λ; *attR*_*λ*_: *attR *site of phage λ; *attB*_*λ*_: *attB *site of phage λ; IPTG: isopropyl-β-D-thiogalactopyranoside; nt: nucleotide; bp: base pair(s); oligos: oligonucleotides; PCR: polymerase chain reaction; ssDNA: single-stranded DNA; dsDNA: double-stranded DNA.

## Authors' contributions

JIK and YH are the project leaders in AGRI and in Ajinomoto, respectively. JIK obtained SC17(0), designed the main experiments concerned with λ Red-driven modifications, and drafted the manuscript. YH developed the transfer of λ Red-driven mutations to *P. ananatis *strains differed from SC17(0). LIG constructed all recombinant plasmids, expressing λ Red genes, tested their function initially in *E*. *coli*, and edited the manuscript. TMK performed the λ Red-driven modifications of the chromosome of *P. ananatis *SC17(0) strain. IGA performed λ Red-driven oligos integration into chromosome of *P. ananatis *SC17 strain. SVM supervised and coordinated the work and edited the manuscript. All authors have read and approved the final version of the manuscript.

## Supplementary Material

Additional file 1 **Plasmids constructed for this study**. Detailed description of plasmids constructed in this study. Click here for file

Additional file 2 **Primers used for this study**. List of primers. Click here for file
