# Supplementary material for: Use of the λ Red-recombineering method for genetic engineering of Pantoea ananatis
Source: BMC Mol Biol. 2009 Apr 23;10:34. doi: 10.1186/1471-2199-10-34 (PMC2682490; doi:10.1186/1471-2199-10-34)
Supplement: Additional file 1 — Plasmids constructed for this study. Detailed description of plasmids constructed in this study. [file 1471-2199-10-34-S1.doc]

pRSFsacB plasmid consists of following fragments: 1) SacI-PstI/BglII fragment corresponds to 7,5 kb fragment of RSF1010 plasmid; 2) PstI/BglII-XbaI fragment contains *E.coli* crp-independent promoter P*_lac_*_UV5_ obtained from pKK233-2; 3) XbaI-XbaI fragment contains *E.coli* *lacI*-repressor gene with changed start-codon (GTG by ATG) and under control of RBS of gene 10 of T7 phage; 4) XbaI-BamHI/BglII contains levansucrase *sacB* gene obtained from chromosome of *B. subtillis*; 5) BamHI/BglII –SacI fragment contains chloramphenicol resistance gene *cat* obtained from pACYC184;

p

p

pRSFPlacsacB plasmid has been obtained from pRSFsacB plasmid by digestion with XbaI restrictase followed by ligation. So, *lacI* gene was eliminated from the plasmid.

p

pRSFRedTER plasmid consists of following fragments: 1) NotI-Ecl136II/EcoRI fragment corresponds to the larger NotI-SacI fragment from RSFsacB plasmid; 2) Ecl136II/EcoRI-BamHI fragment contains terminator of *E.coli rrnB* operon obtained by amplification from MG1655 chromosome and *E.coli* P*_lac_*_UV5_ promoter; 3) PvuI-NotI fragment contains λ Red α, β, and γ genes and transcription terminator tL3 obtained by amplification from pKD46 plasmid;

pRSFRedkan plasmid consist of a two fragments: the first KpnI-HindIII/BamHI fragment corresponds to the largest HindIII-KpnI fragment of pRSFRedTER plasmid, the second fragment contains kanamycin resistance gene from pUC4K and terminator of *E.coli rrnB* operon.

p

p

pRSFGamBet plasmid has the same structure as pRSFRedTER plasmid except the PvuI-NotI fragment, which contains only λ Red α and β genes.

p

pRSFGamBetkan plasmid consist of a two fragments: the first KpnI-HindIII/BamHI fragment corresponds to the largest HindIII-KpnI fragment of pRSFGamBet plasmid, the second fragment contains kanamycin resistance gene from pUC4K and terminator of *E.coli rrnB* operon.
