# Supplementary material for: Use of the λ Red-recombineering method for genetic engineering of Pantoea ananatis
Source: BMC Mol Biol. 2009 Apr 23;10:34. doi: 10.1186/1471-2199-10-34 (PMC2682490; doi:10.1186/1471-2199-10-34)
Supplement: Additional file 2 — Primers used for this study. List of primers. [file 1471-2199-10-34-S2.doc]

Oligonucleotides used for this study

galK-5

5’-cg-cag-ggt-gcc-ggg-tta-agt-tct-tcc-gct-tca-ctg-gtg-aag-cct-gct-ttt-tta-tac-taa-gtt-gg-3'

galK-3

5’-gat-aaa-gct-gct-gca-ata-cgg-ttc-cga-ccg-cga-ctt-cgc-tca-agt-tag-tat-aaa-aaa-gct-gaa-c-3'

galK-t1

5’-gat-gcg-ccc-att-gtc-gca-cat-g-3’

galK-t2

5’-gag-cgc-gga-aat-tag-ctg-atc-3’

galK-F

5’-ccg-cag-ggt-gcc-ggg-tta-agt-tct-tcc-gct-tca-ctg-gaa-gtc-gcg-gtc-gga-acc-gta-ttg-cag-cag-ctt-tat-c-3’

hisD-5

5’-CCA-TAG-CGG-TTG-GAG-ATC-GCA-ATG-CAT-TGC-TGC-ATA-TCC-Ctg-aag-cct-gct-ttt-tta-tac-taa-gtt-gg-3’

hisD-3

5’-gcc-cgc-cag-gca-ctg-gaa-agc-agc-cgc-ctg-atc-gtc-gcc-ccg-ctc-aag-tta-gta-taa-aaa-agc-tga-ac-3’

his-plac-5

5’-CCA-TAG-CGG-TTG-GAG-ATC-GCA-ATG-CAT-TGC-TGC-ATA

-TCC-Cgc-ggg-cag-tga-gcg-caa-cgc-3’

his-cat-3

5’-gcc-cgc-cag-gca-ctg-gaa-agc-agc-cgc-ctg-atc-gtc-gcc-ctt-acg-ccc-cgc-cct-gcc-act-c-3’

hisD-t1

5’-ggc-ata-ggc-cgc-ata-gcg-gg-3’

hisD-t2

5’-ccg-ctg-ttt-tct-acc-gtt-ct-3’

his-XhoI-1

5’-AGC-GGT-TGG-AGA-TCG-CAA-TGC-ATT-GCT-GCA-TAT-CTC-GAG-CGA-CGA-TCA-GGC-GGC-TGC-TTT-CCA-GTG-CCT-GGC-3’

his-XhoI-2

5’-GCC-AGG-CAC-TGG-AAA-GCA-GCC-GCC-TGA-TCG-TCG-CTC-GAG-ATA-TGC-AGC-AAT-GCA-TTG-CGA-TCT-CCA-ACC-GCT-3’

his-SL

5’-ACC-AGA-TCC-CGT-GGC-GTG-CGG-GTT-TGC-AGA-ATC-AGG-TGC-

TCC-GGA-CCA-TAG-CGG-TTG-GAG-ATC-GCA-ATG-C-3’

his-SR

5’-GAG-GCG-GTG-GAG-GAT-CAG-CTG-GCC-CAG-TTG-CCA-

CGT-GCG-GCG-ACA-GCC-CGC-CAG-GCA-CTG-GAA-AGC-AGC-C-3’
